# Incidence of cutaneous melanoma and Merkel cell carcinoma in patients with primary cutaneous B-cell lymphomas: A population study of the SEER registry

**DOI:** 10.3389/fmed.2023.1110511

**Published:** 2023-04-06

**Authors:** Lauren Banner, Daniel Joffe, Emily Lee, Pierluigi Porcu, Neda Nikbakht

**Affiliations:** ^1^Department of Dermatology and Cutaneous Biology, Thomas Jefferson University, Philadelphia, PA, United States; ^2^Division of Hematologic Malignancies and Hematopoietic Stem Cell Transplantation, Department of Medical Oncology, Thomas Jefferson University, Philadelphia, PA, United States

**Keywords:** primary cutaneous B-cell lymphoma (PCBCL), primary cutaneous follicle center lymphoma (PCFCL), cutaneous melanoma (CM), Surveillance, Epidemiology, and End Results (SEER), Merkel cell carcinoma (MCC), primary cutaneous marginal zone lymphoma (PCMZL)

## Abstract

**Introduction:**

The increased incidence of cutaneous melanoma (CM) and Merkel cell carcinoma (MCC) in patients with hematologic malignancies (HM) is well established. While the risk of CM has been assessed in some subtypes of HM including cutaneous T-cell lymphoma, the incidence in patients with primary cutaneous B-cell lymphoma (PCBCL) has not been interrogated.

**Methods:**

Here we evaluated the standardized incidence ratio (SIR) of CM and MCC in 5,179 PCBCL patients compared to approximately 1.5 billion individuals in the general population using the Surveillance, Epidemiology and End Results (SEER) database. Among patients with PCBCL, we identified subgroups that were at increased risk for CM or MCC as a second primary cancer.

**Results:**

We found 36 cases of CM in the PCBCL cohort (SIR, 1.35; 95% CI, 0.94–1.86), among which SIR was significantly elevated for non-Hispanic White patients compared to the general population (SIR, 1.48; 95% CI, 1.03–2.06). Males had a significantly increased risk of developing CM after a diagnosis of PCBCL (SIR, 1.60; 95% CI, 1.10–2.26). We found that males in the age group of 50–59 were at increased risk for CM development (SIR, 3.02; 95% CI, 1.11–6.58). Males were at increased risk of CM 1–5 years after PCBCL diagnosis (SIR, 2.06; 95% CI, 1.18–3.34). Patients were at greater risk of developing MCC within 1 year of diagnosis of PCBCL (SIR, 23.60; 95% CI, 2.86–85.27), particularly in patients who were over the age of 80 (SIR, 46.50; 95% CI, 5.63–167.96). Males aged 60–69 with PCBCL, subtype marginal zone, were also at increased risk for MCC (SIR, 42.71; 95% CI, 1.08–237.99).

**Conclusion:**

There is an increased incidence of CM in White, middle-aged males within 5 years of diagnosis of PCBCL and an increased risk of MCC in elderly patients within 1 year of PCBCL diagnosis. These data suggest that certain subgroups of patients with PCBCL may require more rigid surveillance for CM and MCC.

## Introduction

Cutaneous melanoma (CM) is the fifth most common type of cancer and represents 5.2% of the cancers diagnosed in the United States. CM is responsible for 1.3% of all cancer deaths and is most prevalent in non-Hispanic White males between the ages 65–74 (1). The age-adjusted rate of CM is 22.2 per 100,000 as calculated by SEER*Stat software package 8.4.0.1 (National Cancer Institute, Bethesda, MD). Merkel cell carcinoma (MCC) is a rare aggressive cutaneous neuroendocrine tumor that is common in older white males with an age-adjusted rate of 0.6 per 100,000. An increased association of CM in patients with hematologic malignancies (HM) has been reported (Table 1) (2–28). MCC incidence has not been as widely studied in patients with HM, but it has been consistently reported to develop at an increased incidence in patients with HM (Table 1) (5, 24–33). When examining all subtypes of HM, over two thirds of cohorts exhibited a significantly increased risk of CM occurring after an HM diagnosis (Table 1) (2, 3, 5, 6, 8, 9, 11, 12, 14–19, 21, 34). Three studies examined the development of second primary solid malignancies in cutaneous T-cell lymphomas (CTCL), two of which found up to a 9-fold increase in the risk of second primary CMs (9, 10, 35).

**TABLE 1:** Epidemiologic studies for CM and MCC as subsequent primary malignancy after HM (2, 3, 5, 6, 8, 9, 11, 12, 14, 15, 17–19, 21, 30, 31, 33–35).

Reference	Location	Type of lymphoma	Number of patients	Number of CMs after lymphoma diagnosis	Relative risk or standardized incidence ratio	95% confidence interval or *p*-value
**CM occurring after diagnosis of HM**						
Travis et al. (17)	United States	NHL	29,153	44	*2.44* *	1.78–3.28
Travis et al. (18)	United States	CLL	9,456	28	2.79*	1.85–4.03
Adami et al. (2)	Denmark, Sweden	NHL	34,641	52	2.4*	1.8–3.2
		CLL	17,400	34	3.1*	2.1–4.4
Dong and Hemminki (21)	Sweden	NHL	18,960	33	1.14	0.78–1.60
Goggins et al. (8)	United States	NHL	62,597	139	1.75*	1.48–2.07
McKenna et al. (14)	Scotland	NHL	13,857	18	2.1*	1.2–3.6
		CLL	4,016	6	2.3	0.0–2.4
Hisada et al. (11)	United States	CLL	16,367	90	3.18*	*p* < 0.05
Brennan et al. (34)	International	NHL	109,451	258	1.92*	1.69–2.16
Huang et al. (12)	United States	CTCL	1,798	10	2.60*	1.25–4.79
Brownell et al. (35)	United States	CTCL	672	2	2.82	0.34–10.18
Tsimberidou et al. (19)	United States	CLL/SLL	2,028	19	6.17*	3.97–9.24
Brewer et al. (5)	United States	CLL	28,964	268	2.0*	1.8–2.2
		NHL	94,967	441	1.5*	1.4–1.6
Chang et al. (6)	United States	MM	31,622	63	1.27	0.97–1.62
Archibald et al. (3)	United States	CLL	470	22	6.32*	3.45–10.60
Goyal et al. (9)	United States	CTCL < 1 year	6,742	4	9.61*	2.62–24.62
		CTCL > 1 year		20	9.0*	5.5–13.90
Singh et al. (15)	United States	MM	79,174	280	1.26*	*p* < 0.001
**MCC occurring after diagnosis of HM**						
Howard et al. (33)	United States	CLL	17,315	14	6.89*	3.77–11.57
		NHL	81,743	16	3.37*	1.93–5.47
		MM	23,949	4	3.70*	1.01–9.47
Koljonen et al. (30)	United States	CLL	4,164	4	15.7*	3.2–46.0
Koljonen et al. (31)	Nordic Countries	NHL	109,838	18	4.34*	2.57–6.85
Brewer et al. (5)	United States	CLL	28,964	31	8.2*	5.6–11.6
		NHL	94,967	23	5.9*	3.8–8.9

CM, cutaneous melanoma; MCC, Merkel cell carcinoma; HM, hematologic malignancy; PCBCL, primary cutaneous B-cell lymphoma. Asterisk (*), bold, italics indicates *p* < 0.05.

Primary cutaneous B-cell lymphomas (PCBCL) are subtypes of HM that represent one quarter of all cutaneous lymphomas. The incidence of PCBCL is <1/100,000 person years and increases with age. The two most common types of PCBCL are primary cutaneous marginal zone lymphoma (PCMZL) and primary cutaneous follicle center lymphoma (PCFCL) which are typically indolent. Primary cutaneous diffuse large B-cell lymphoma, leg type (PC-DLBCL, LT) tends to be the most aggressive (36, 37). No previous studies have examined the incidence of CM or MCC in PCBCL. We endeavored to analyze the incidence of CM and MCC in the PCBCL population compared the general population using the Surveillance, Epidemiology and End Results Projects (SEER) database.

## Materials and methods

### SEER patient cohort selection

The SEER cohort selection of patients with PCBCL was adapted from Bomze et al. (38). Patients were diagnosed between January 2000 and December 2019 in 17 cancer registries that included San Francisco, Connecticut, Hawaii, Iowa, New Mexico, Seattle (Puget Sound), Utah, Atlanta (Metropolitan), San Jose-Monterey, Los Angeles, Alaska Natives, Rural Georgia, California excluding SF/SJM/LA, Kentucky, Louisiana, New Jersey and Greater Georgia [SEER 17 Regs excluding AK Research Data, November 2021 Sub (2000–2019)]. Patients with PCBCL were identified according to the World Health Organization’s International Classification of Diseases for Oncology, third edition (ICD-O-3) by the following histological codes: diffuse large B-cell lymphoma (ICD-O-3 9680); follicular lymphoma (ICD-O-3 9690), follicular lymphoma grades 1–3 (ICD-O-3 9691, 9695, 9698), extranodal marginal zone lymphoma of mucosal associated lymphoid tissue-MALT (ICD-O-3 9699), Non-Hodgkin Lymphoma, NOS (ICD-O-3 9591), B-cell lymphoma, between diffuse large B and HL (ICD-O-3 9596), Primary cutaneous follicle center lymphoma (ICD-O-3 9597), large B, diffuse, immunoblastic (ICD-O-3 9684). We included cases with the topography code for skin (C44) as the primary site. PCBCL was selected as the first primary cancer. The outcome event variables were selected using the Site recode ICD-O-3/WHO 2008 (for SIRs) codes for CM and MCC. The timing of the first primary diagnosis of PCBCL was used as the initial date from which CM and MCC latency were calculated. Patients were excluded if the report was obtained solely from a death certificate or autopsy report with no confirmation of diagnosis. Study of this SEER cohort was exempt from institutional review board approval.

### Statistical analysis

The measure of relative risk was estimated as the standardized incidence ratio (SIR), the ratio of observed cases to expected cases of CM or MCC (O/E) in the SEER cohort. An SIR of 1 indicated no difference in incidence compared to the general population. The statistical significance of SIR was assessed using a 95% confidence interval (CI).

To calculate the number of expected CMs and MCCs, a reference rate file of CM per 100,000 was calculated using SEER*Stat software package 8.4.0.1 (National Cancer Institute, Bethesda, MD) and applied to the number of patients in the SEER-17 registry of 1,628,926,957 persons. In the SEER cohort, the number of person-years at risk among patients from diagnosis of PCBCL to a second diagnosis of CM or MCC was calculated by SEER*Stat software package 8.4.0.1 (National Cancer Institute, Bethesda, MD, USA).

## Results

### Patients from SEER cohort

The SEER PCBCL cohort consisted of 5,179 patients of whom 36 (0.70%) received a diagnosis of CM (SIR, 1.35; 95% CI, 0.94–1.86) and three (0.06%) received a diagnosis of MCC (SIR, 3.74 95% CI 0.77–10.92). The median age range of developing the subsequent CM was 70–79. Two patients who developed MCC were over 85 years old, and the other patient was 69. There were no cases of subsequent CM or MCC diagnosed before the age of 40. CM occurred most frequently in PCBCL patients over age 80. The most frequent latency period for CM to appear was between 1 and 5 years after PCBCL diagnosis. Two second primary MCCs occurred within a year and one MCC occurred 1–5 years after PCBCL diagnosis. Eleven CMs were reported as stage I and the remaining cases did not have a stage reported. All the second primary MCCs were localized.

Thirty-five of the thirty-six CMs in patients with PCBCL occurred in non-Hispanic White persons (97%). The SIR showed a significant elevation at 1.48; 95% CI, 1.03–2.06. Only one CM occurred in the non-White group (3%). This patient’s race was reported as Asian or Pacific Islander. All patients who developed second primary MCC after PCBCL diagnosis were white.

### Risk of second primary CM and MCC as a function of sex, attained age and latency

Males with PCBCL comprised 57% of the cohort (*n* = 2,973) and females comprised 43% (*n* = 2,206). Thirty-two males (1.1%) and four females (0.18%) developed CM. Two males (0.06%) and 1 female (0.04%) developed MCC. When comparing gender, males had a significantly increased risk of developing CM after a diagnosis of PCBCL (SIR, 1.60; 95% CI, 1.10–2.26). Whereas females were not at an increased risk of developing CM (SIR, 0.59; 95% CI, 0.16–1.51). Males in the age groups 50–59 were the most at risk of CM (SIR, 3.02; 95% CI, 1.11–6.58) (Table 2). Males were also at an increased risk of CM between 1 and 5 years after diagnosis of PCBCL (SIR, 2.06; 95% CI, 1.18–3.34) (Table 3). For males, the age group 60–69 was most at risk for CM when accounting for the 1–5-year latency period (SIR, 3.07; 95% CI, 1.13–6.68). Patients with PCBCL were more at risk for developing MCC within a year of their lymphoma diagnosis (SIR, 23.60; 95% CI, 2.86–85.27) (Table 3). Patients who were 80 or older were at an increased risk of developing MCC with a year of diagnosis of PCBCL (SIR, 46.50; 95% CI, 5.63–167.96). Females over 80 years old were at an increased risk for MCC within 1 year of PCBCL diagnosis (SIR, 66.06; 95% CI, 1.67–368.06).

**TABLE 2:** Age and gender specific instances of CM and MCC.

	Gender					
Age	CM			MCC		
	All SIR (95% CI)	Male SIR (95% CI)	Female SIR (95% CI)	All SIR (95% CI)	Male SIR (95% CI)	Female SIR (95% CI)
40–49	2.32 (0.28–8.39)	3.53 (0.43–12.74)	0 (0–12.56)	0 (0–1,065.17)	0 (0–1,409.08)	0 (0–4,364.30)
50–59	2.15 (0.79–4.68)	3.02* (1.11–6.58)	0 (0–4.59)	0 (0–127.41)	0 (0–167.97)	0 (0–527.60)
60–69	1.38 (0.63–2.61)	1.44 (0.58–2.96)	1.20 (0.15–4.33)	8.78 (0.22–48.93)	11.67 (0.30–65.04)	0 (0–130.76)
70–79	0.93 (0.40–1.84)	1.20 (0.52–2.36)	0 (0–1.95)	0 (0–14.90)	0 (0–20.14)	0 (0–57.27)
80+	1.42 (0.71–2.54)	1.58 (0.72–3.00)	0.98 (0.12–3.55)	4.90 (0.59–17.69)	3.59 (0.09–19.99)	7.70 (0.19–42.91)
Total	1.35 (0.94–1.86)	1.60* (1.10–2.26)	0.59 (0.16–1.51)	3.74 (0.77–10.92)	3.49 (0.42–12.62)	4.34 (0.11–24.19)

CM, cutaneous melanoma; MCC, Merkel cell carcinoma; PCBCL, primary cutaneous B-cell lymphoma; SIR, standardized incidence ratio; CI, confidence interval. Asterisk (*), bold, italics indicates *p* < 0.05.

**TABLE 3:** Patient latency to diagnosis of CM and MCC from PCBCL stratified by gender.

	Gender					
Latency (months)	CM			MCC		
	All SIR (95% CI)	Male SIR (95% CI)	Female SIR (95% CI)	All SIR (95% CI)	Male SIR (95% CI)	Female SIR (95% CI)
<12	0.73 (0.09–2.64)	0.51 (0.01–2.82)	1.31 (0.03–7.30)	23.60* (2.86–85.27)	17.15 (0.43–95.56)	37.85 (0.96–210.87)
12–59	1.60 (0.93–2.57)	2.06* (1.18–3.34)	0.35 (0.01–1.97)	3.11 (0.08–17.32)	4.43 (0.11–24.70)	0 (0–38.38)
60–119	1.10 (0.50–2.08)	1.30 (1.18–3.34)	0.49 (0.01–2.72)	0 (0–15.19)	0 (0–21.24)	0 (0–53.33)
120–179	1.71 (0.69–3.52)	1.90 (0.70–4.13)	1.08 (0.03–5.99)	0 (0–30.61)	0 (0–41.22)	0 (0–118.91)
180+	0.89 (0.02–4.97)	1.13 (0.03–6.27)	0 (0–15.91)	0 (0–112.20)	0 (0–146.16)	0 (0–482.91)
Total	1.35 (0.94–1.86)	1.60* (1.10–2.26)	0.59 (0.16–1.51)	3.74 (0.77–10.92)	3.49 (0.42–12.62)	4.34 (0.11–24.19)

CM, cutaneous melanoma; MCC, Merkel cell carcinoma; PCBCL, primary cutaneous B-cell lymphoma; SIR, standardized incidence ratio; CI, confidence interval. Asterisk (*), bold, italics indicates p < 0.05.

### The risk of second primary CM and MCC by type of PCBCL

Of the three most common types of PCBCL, there were 13 cases of CM in 1,844 PCFCL patients (0.70%) with an SIR of 1.25 (95% CI, 0.67–2.14). All patients were male (Table 4). There was 1 MCC case found in a male PCFCL patient (SIR, 3.31; 95% CI 0.08–18.42) (Table 4). Eleven CMs were in 1,641 PCMZL patients (0.67%) with an SIR of 1.49 (95% CI, 0.74–2.66) (Table 4). One MCC occurred in a male patient after diagnosis of PCMZL (SIR, 5.03; 95% CI 0.13–28.03) (Table 4). There were four patients with CM occurring after diagnosis of PC-DLBCL (*n* = 1,235; 0.32%) with an SIR of 0.66 (95% CI, 0.18–1.69) (Table 4). These patients were all male. There was one female patient who developed MCC after diagnosis of PC-DLBCL (SIR, 4.83; CI 95% 0.12–26.89) (Table 4). The remaining second primary CMs occurred in patients with other rare PCBCLs and in patients with PCBCL not specified. When accounting for age, gender, and subtype, the SIR was significantly increased for males 50–54 with PCFCL to develop CM (SIR, 8.31; 95% CI, 1.01–30.03). Males in the age group 60–69 with the subtype of PCMZL were at an increased risk for MCC (SIR, 42.71; 95% CI 1.08–237.99).

**TABLE 4:** Incidence of CM and MCC in PCBCL stratified by type, age and gender.

		Gender					
Subtype	Age	CM			MCC		
		All SIR (95% CI)	Male SIR (95% CI)	Female SIR (95% CI)	All SIR (95% CI)	Male SIR (95% CI)	Female SIR (95% CI)
PCFCL	40–49	0 (0–13.38)	0 (0–21.33)	0 (0–35.88)	0 (0–3,377.67)	0 (0–4,514.28)	0 (0–13,415.15)
	50–59	2.99 (0.62–8.74)	4.11 (0.85–12.01)	0 (0–13.48)	0 (0–349.61)	0 (0–450.59)	0 (0–1,560.01)
	60–69	1.13 (0.23–3.30)	1.46 (0.30–4.26)	0 (0–6.13)	0 (0–80.03)	0 (0–102.35)	0 (0–366.90)
	70–79	0.84 (0.17–2.46)	1.04 (0.21–3.03)	0 (0–5.51)	0 (0–36.79)	0 (0–47.28)	0 (0–165.81)
	80+	1.42 (0.39–3.65)	1.92 (0.52–4.90)	0 (0–5.13)	6.90 (0.17–38.45)	10.14 (0.26–56.49)	0 (0–79.71)
	Total	1.25 (0.67–2.14)	1.63 (0.87–2.78)	0 (0–1.54)	3.30 (0.08–18.39)	4.51 (0.11–25.13)	0 (0–45.41)
PCMZL	40–49	2.51 (0.06–13.98)	3.91 (0.10–21.76)	0 (0–25.87)	0 (0–2,414.04)	0 (0–3,334.02)	0 (0–8,748.48)
	50–59	1.04 (0.03–5.77)	1.61 (0.04–8.97)	0 (0–10.69)	0 (0–383.75)	0 (0–550.41)	0 (0–1,267.38)
	60–69	1.54 (0.32–4.50)	1.50 (0.18–5.41)	1.63 (0.04–9.07)	29.78 (0.75–165.92)	42.71* (1.08–237.99)	0 (0–362.80)
	70–79	0.93 (0.11–3.36)	1.23 (0.15–4.43)	0 (0–7.13)	0 (0–60.18)	0 (0–83.82)	0 (0–213.44)
	80+	2.24 (0.61–5.74)	1.52 (0.18–5.50)	4.23 (0.51–15.29)	0 (0–39.85)	0 (0–57.49)	0 (0–129.85)
	Total	1.49 (0.74–2.66)	1.52 (0.66–3.00)	1.39 (0.29–4.06)	5.03 (0.13–28.03)	7.17 (0.18–39.93)	0 (0–62.27)
DLBCL	40–49	0 (0–30.85)	0 (0–43.70)	0 (0–104.87)	0 (0–6,757.31)	0 (0–8,441.33)	0 (0–33,871.90)
	50–59	1.81 (0.05–10.10)	2.31 (0.06–12.88)	0 (0–30.99)	0 (0–622.03)	0 (0–763.79)	0 (0–3,351.45)
	60–69	0.76 (0.02–4.22)	0.98 (0.02–5.49)	0 (0–12.08)	0 (0–156.67)	0 (0–204.56)	0 (0–669.20)
	70–79	0 (0–1.96)	0 (0–2.74)	0 (0–6.84)	0 (0–63.54)	0 (0–94.77)	0 (0–192.80)
	80+	0.93 (0.11–3.35)	1.32 (0.16–4.76)	0 (0–5.78)	8.40 (0.21–46.79)	0 (0–47.76)	23.90 (0.61–133.17)
	Total	0.66 (0.18–1.69)	0.91 (0.25–2.32)	0 (0–2.23)	4.83 (0.12–26.89)	0 (0–26.45)	14.77 (0.37–82.30)

CM, cutaneous melanoma; MCC, Merkel cell carcinoma; PCBCL, primary cutaneous B-cell lymphoma; SIR, standardized incidence ratio; CI, confidence interval; PCFCL, primary cutaneous follicle center lymphoma; PCMZL, primary cutaneous marginal zone lymphoma; DLBCL, diffuse large B-cell lymphoma. Asterisk (*), bold, italics indicates *p* < 0.05.

## Discussion

This study is the first to define the relationship between PCBCL and the incidence of CM and MCC. In the SEER cohort, we were able to identify at-risk populations among PCBCL patients. The incidence ratio for developing CM after diagnosis of PCBCL was significantly increased in males but not in females. The risk of acquiring CM was higher between 1 and 5 years of a diagnosis of PCBCL. Females over the age of 80 with a diagnosis of PCBCL were at an increased risk of developing MCC within 1 year of diagnosis. Males in the age groups 50–54 were at particularly increased risk for CM, especially with the subtype PCFCL. Subtype PCMZL carried the highest risk of MCC for males aged 60–69. These observations highlight the importance of vigilant monitoring of these populations for a second primary skin cancer.

When CM is caught early, it is highly treatable with a 99% 5-year survival for localized disease compared to a 32–52% 5-year survival for distant stage CM (1, 39, 40). MCC is also treatable with an 81% 5-year survival for stage 1 disease compared to an 11% 2-year survival rate for stage 4 disease (4). There is a lack of national consensus on the screening guidelines for CM and non-melanoma skin cancers. The United States Prevention Services Task Force (USPSTF) cites insufficient evidence to recommend screenings for asymptomatic persons. However, both the USPSTF and the American Academy of Dermatology (AAD) recommend that dermatologists examine high-risk individuals more frequently. It is therefore crucial to define risk factors for CM and MCC, so that surveillance opportunities are not missed. Based on our findings, patients with PCBCL, specifically PCFCL or PCMZL, who are middle-aged to elderly can benefit from annual total body skin exams (TBSEs) within 5 years of diagnosis of cutaneous B-cell lymphoma.

Most prior studies found a highly increased risk for CM and MCC development in hematologic malignancy (Table 1). Several mechanisms may contribute to the increased risk of CM and MCC in patients with hematologic malignancies, including their immunocompromised status (4, 13, 41). A bidirectional relationship has been noted in prior studies between HM and CM or MCC (2, 7, 20, 30, 31, 33). Another factor that may be contributing to the development of MCC and CM in patients with hematologic malignancy is receiving immunosuppressive treatments such as radiation (a commonly used treatment modality in PCFCL and PCMZL) and chemotherapy (13, 23). Unfortunately, using data available in SEER, we were not able to determine if the sites of MCC and CM were the same sites previously radiated.

The two most common subtypes of PCBCL are PCMZL and PCFCL. They are more indolent than their nodal counterparts, with 5-year disease specific survival rates of 99 and 95%, respectively (37). PC-DLBCL, LT is a less common but more aggressive subtype of PCBCL with a disease specific survival rate of 40–60% (37, 41). Interestingly, only indolent PCFCL and PCMZL had increased SIRs for CM and MCC, respectively, implying that aggressive molecular features in PCBCL may not drive pre-disposition to CM or MCC. Ultimately, mechanisms that lead to the development of CM and MCC after cutaneous B-cell lymphomas remain to be elucidated. Overall, indolent PCBCLs require monitoring for both recurrence of cutaneous lymphoma as well as CM and MCC.

There were several limitations to our study. Firstly, the cohorts were reviewed retrospectively instead of prospectively, and given the rarity of PCBCL, the cohort in SEER was small. Coding for primary cutaneous B-cell lymphoma is a multi-step process which may lead to cases being missed in the registry. Additionally, patients with PCBCL may be seen more frequently at dermatology offices rather than oncology practices, where SEER data entry is commonly performed. Another limitation was that we were unable to account for family history as a risk factor for these malignancies using the SEER data. As the occurrences of MCC and PCBCL are very rare, it is likely that larger sample sizes may be needed to assess correlations. We recommend future large, multi-institutional prospective studies on the risk of CM and MCC in patients with PCBCL.

## Data availability statement

The raw data supporting the conclusions of this article will be made available by the authors, without undue reservation.

## Ethics statement

Ethical review and approval was not required for the current study in accordance with the local legislation and institutional requirements. Written informed consent for participation was not required for this study in accordance with the national legislation and the institutional requirements.

## Author contributions

LB participated in the data analysis and wrote the manuscript. DJ contributed to the data collection and edited the manuscript. EL contributed to the statistical analysis and wrote the manuscript. PP and DJ edited the manuscript. NN participated in the research design, editing, and supervised the production of the manuscript. All authors contributed to the article and approved the submitted version.
